# Are we missing non-motor seizures in Parkinson’s disease? Two case reports

**DOI:** 10.1186/s40734-017-0061-3

**Published:** 2017-09-05

**Authors:** Andre Y. Son, Alberto Cucca, Shashank Agarwal, Anli Liu, Alessandro Di Rocco, Milton C. Biagioni

**Affiliations:** 1 The Marlene and Paolo Fresco Institute for Parkinson’s & Movement Disorders, NYU Langone Medical Center – New York University School of Medicine, New York, NY USA; 2 NYU Comprehensive Epilepsy Center, NYU Langone Medical Center – New York University School of Medicine, New York, NY USA; 3 The Marlene and Paolo Fresco Institute for Parkinson’s & Movement Disorders, NYU Langone Medical Center, 240 East 38th Street, 20th Floor, New York, NY 10016 USA

**Keywords:** Parkinson’s disease, Non-motor symptoms, Non-motor seizures, Cognitive changes

## Abstract

**Background:**

Parkinson’s disease (PD) is predominantly recognized for its motor symptoms, but patients struggle from a morbid and heterogeneous collection of non-motor symptoms (NMS-PD) that can affect their quality of life even more. NMS-PD is a rather generalized term and the heterogeneity and non-specific nature of many symptoms poses a clinical challenge when a PD patient presents with non-motor complaints that may not be NMS-PD.

**Case presentation:**

We report two patients with idiopathic PD who presented with acute episodes of cognitive changes. Structural brain images, cardiovascular and laboratory assessment were unremarkable. Both patients experienced a considerable delay before receiving an epilepsy-evaluation, at which point electroencephalogram abnormalities supported the diagnosis of focal non-motor seizures with alteration of awareness. Antiepileptic therapy was implemented and was effective in both cases.

**Conclusions:**

Diagnosing non-motor seizures can be challenging. However, PD patients pose an even greater challenge given their eclectic non-motor clinical manifestations and other disease-related complications that could confound and mislead adequate clinical interpretation. Our two cases provide examples of non-motor seizures that may mimic non-motor symptoms of PD. Treating physicians should always consider other possible causes of non-motor symptoms that may coexist in PD patients. Epilepsy work-up should be contemplated in the differential of acute changes in cognition, behavior, or alertness.

## Background

There is a growing recognition and interest of non-motor symptoms of Parkinson’s disease (NMS-PD). In fact, NMS-PD have been shown to affect patients’ quality of life even more than the motor symptoms [1, 2]. A broad and heterogeneous collection of NMS-PD has been observed and groups have described various phenotypes using patterns of NMS-PD [3, 4]. Many NMS-PD are non-specific, can precede motor symptoms, and become more prominent in advanced PD. As such, it can be a challenge to determine if a non-motor feature is actually a part of the NMS-PD or due to another underlying condition. Moreover, some NMS-PD can present acutely and resolve in a recurrent episodic fashion. We report two patients with PD who developed recurrent focal non-motor features that were ultimately diagnosed with non-motor seizures to raise awareness of this particular challenge.

## Case presentation

This retrospective chart review was exempt for consent from the local institutional review board. Both patients had a confirmed diagnosis of idiopathic PD established by a Movement Disorders specialist. Neither patient had a personal or family history of epilepsy. Neither patient had a history of deep brain stimulation, traumatic brain injury, stroke, or other brain structural abnormality.

### Case 1

A 64-year-old left-handed woman developed right-leg shuffling and right-hand tremor. She was then diagnosed with idiopathic PD and treated in our movement disorder clinic. At age 70, she started experiencing episodes of protracted confusion characterized mostly by mild forgetfulness and disorientation episodes that lasted few hours along with frequent nocturnal events (violent jerks and screaming). At age 72, after waking up one morning, she was discovered confused, staring, not oriented to place or time, unable to recall large periods of the previous day. This time she was taken to the emergency room. After ruling out metabolic disorders, infections, orthostatic hypotension, and an unremarkable brain MRI, she was discharged with the diagnosis of transient global amnesia (TGA). At age 73, she had an episode of protracted confusion preceded by visual perception of “grid patterns” in bilateral visual fields and an olfactory hallucination though she had been anosmic for many years. She was taken to the emergency room and admitted for further workup. She did not have headache or any focal neurological symptom or focal deficit. Head CT and brain MRI were unremarkable and orthostatic hypotension workup was negative. Two different electroencephalograms (EEGs) were obtained in the following year. One showed independent bilateral temporal spikes and, the second one, left temporal slowing and left anterior epileptiform sharp waves. After the first EEG, she was diagnosed with focal onset epilepsy. Her epileptologist prescribed lamotrigine and a few months later added levetiracetam. The confusional episodes, as well as the nocturnal events, completely resolved. However, despite adequate seizure control, her PD progressed quite rapidly with severe motor deterioration, balance impairment with falls, troublesome dyskinesias and dystonia episodes. She also developed psychiatric manifestations (delusions, hallucinations), sleep disorder and cognitive fluctuations. By her last visit (at age 79), she was seizure free but entirely dependent for her activities of daily living (ADLs) and had moderate dementia.

### Case 2

**Fig. 1:**
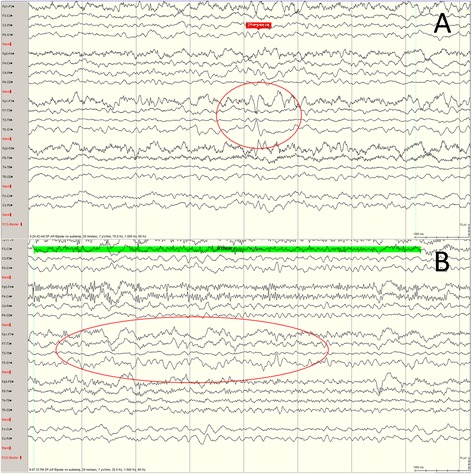
Electroencephalogram of *Case 2* prior to antiepileptic therapy. **a** Left temporal sharp wave; **b** Left temporal slowing

**Fig. 2:**
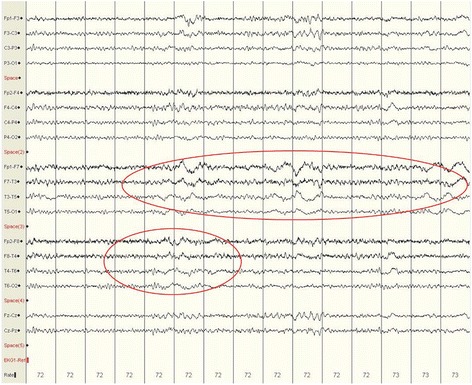
Electroencephalogram of Case 2. One year post-antiepileptic therapy; right and left temporal slowing

A 76-year-old right-handed woman initially presented with asymmetric tremor of hands, bradykinesia and limb rigidity. She was diagnosed with idiopathic PD and was treated in our movement disorder clinic. She had no cognitive, sleep, or psychiatric impairments at the time and was independent with her ADLs. At age 82, she began experiencing episodes of protracted confusion and unresponsiveness without recollection of her actions. The episodes lasted anywhere from minutes to hours and she had no focal neurological symptoms or deficits. Over the course of a year, after several office visits and ruling out common causes of acute-onset cognitive changes, such as metabolic abnormalities, stroke, orthostatic hypotension, infections etc., she was evaluated for possible seizures. A video-EEG demonstrated left temporal sharp wave and focal left temporal slowing (Fig. 1a and b). She was diagnosed with focal non-motor seizures with altered awareness and was treated with levetiracetam. Repeat EEG 1 year following initiation of antiepileptic therapy is shown in Fig. 2. The seizure frequency was reduced to one a year for the next 3 years. At her last visit at age 86, she was seizure-free for 1 year, had mild gradual cognitive decline, but remained independent with her ADLs and without any significant PD motor or non-motor complications.

## Discussion and conclusions

Here we presented two cases of acute onset, recurrent episodes of confusion due to focal non-motor seizures with alteration of awareness. Despite continuous medical follow-up, both cases experienced a considerable delay to diagnosis and treatment of epilepsy. Case 1 had a delay of 3 years and experienced a faster decline of PD motor and non-motor symptoms with severe disability and dementia while Case 2 had a delay of 1 year.

In the elderly, non-motor seizures and non-convulsive status epilepticus are common and their clinical suspicion and diagnosis has always been challenging [5]. A high level of clinical suspicion and supportive electroencephalographic evidence are required to confirm the diagnosis and help to differentiate presentations from other conditions (e.g. transient global amnesia, acute confusional state or delirium).

It is not uncommon for PD patients to have NMS-PD such as cognitive impairment, vivid or violent dreams, paresthesias, visual hallucinations, orthostatic hypotension and syncope, which generate an extra layer of complexity to the clinical interpretations and the differential diagnosis of these acute recurrent episodes (Table 1). To add to the challenge, acute fluctuations of cognition and/or alertness could be the result of diffuse Lewy Body disease [6], a complication of PD characterized by these recurrent episodes. Moreover, other etiologies such as infections, metabolic abnormalities, pharmacological side effects, or vascular events, could elicit an acute confusional state in this vulnerable elderly population [7]. All these factors could confound clinical interpretation and further workup, ultimately delaying or entirely missing an epilepsy diagnosis. While the diagnosis of comorbid epilepsy was made, the etiology of epilepsy in our cases is presently unknown; therefore, our two cases should be considered epilepsy of unknown cause.

**Table 1:** Clinical phenomenology of Cases 1 and 2 Vs common PD-related non-motor symptoms

Case 1 Episodic Manifestations	Case 2 Episodic Manifestations	PD Non-Motor Manifestation
Confusion (few hours duration)	Confusion (minutes to hours duration)	Cognitive fluctuations (hours to days)
Visual aura	-	Visual perceptual problems/hallucinations
Phantosmia	-	Hyposmia/functional anosmia
Nocturnal events (violent jerks and vocalizations)	-	-REM sleep behavior disorder (possible pre-motor) -Non-REM parasomnias (confusional wandering)
EEG: bilateral temporal spikes; left temporal slowing and left anterior sharp waves	EEG: left temporal slowing and left temporal sharp wave	EEG: normal/global slowing

In conclusion, when evaluating PD patients with acute cognitive/behavioral complaints, particularly episodes of confusion, we suggest that clinicians consider non-motor seizures and non-convulsive status epilepticus earlier in the differential diagnosis. Electroencephalography work-up should be promptly conducted to aid clinical interpretation. A prompt diagnosis and adequate treatment may influence PD progression and avoid potentially dangerous complications of delaying or missing diagnosis.

## Abbreviations

ADL: Activities of daily living
EEG: Electroencephalogram
MRI: Magnetic resonance imaging
NMS-PD: Non-motor symptoms of Parkinson’s disease
PD: Parkinson’s disease
TGA: Transient global amnesia
Video-EEG: Video-electroencephalogram
